# Effects of Lighting Quality on Working Efficiency of Workers in Office Building in Tanzania

**DOI:** 10.1155/2019/3476490

**Published:** 2019-11-14

**Authors:** Justine Mushobozi Katabaro, Yonghong Yan

**Affiliations:** ^1^Faculty of Architecture and Urban Planning, Chongqing University, Shapingba District, Chongqing 400045, China; ^2^Key Laboratory of the Ministry of Education of Mountainous City and Towns Construction and New Technology, Chongqing University, Shapingba District, Chongqing 400045, China

## Abstract

**Background:**

In this era of Information Communication Technology, a high-quality working environment is essential to the occupants. Providing quantity rather the quality of work environments is very common in most of the least developed countries, including Tanzania. Existing research asserts that poor indoor environmental quality such as lighting has a detrimental effect on human health, and in case of the office working population, it also affects their work performance. This study aims to analyze the effects of the lighting quality on working efficiency of workers in Tanzania.

**Methods:**

Four representative offices from the administration building at Mbeya University of Science and Technology were investigated from June to September 2018. The customized questionnaire survey tool was administered to the randomly selected occupants to survey their perceptions about the quality of lighting in their workplace and its influence on their health and work efficiency. Physical observation and illuminance distribution measurements were also conducted.

**Results:**

The statistical analysis indicates that the majority of the occupants are less satisfied with the lighting quality in their working environment, and some respondents reported that it significantly affected their work efficiency and wellbeing. The average desk illuminance and uniformity level were found to be below the recommended values of the Chartered Institution of Building Services Engineers (CIBSE) and the International Commission on lighting (CIE).

**Conclusion:**

Despite the suggested improvement measures, this research emphasizes that poorly articulated work environment can adversely affect the productivity and work efficiency of the workers. The workers in such condition are also exposed to occupational diseases. Thus, providing a healthy work environment should be a fundamental right of the workers.

## 1. Introduction

For the past two decades, most of the sub-Saharan African countries have recorded unprecedented economic development and population growth. Because of resource constraints, these achievements have forced some countries to maximize the use of available infrastructures and resources to meet the demand of the increased population [1]. For example, in Tanzania, some existing schools and other institutions' buildings such as Mbeya University of Science and Technology (MUST) were remodeled from their original function to accommodate the new demand of an increased number of staff members and students [2, 3]. The remodeling items included only the increment of offices and classrooms within the existing structures while excluding other demands such as sustainable lighting, acoustics control, thermal condition, and other factors which are very important for improving occupants' satisfaction, morale, well-being, and work productivity [4–7].

The advancement and wide adoption of Information Communication Technology (ICT) has made video display terminals (VDT) the standard working tools in offices [8–10]. These VDT gadgets have almost replaced traditional clerical works. Office spatial quality and environmental quality requirements, such as lighting, have also changed [11]. In connection to this, most of the developed and some of the fast-developing countries have raised the awareness of VDT offices luminous environment requirements through education and by enacting new regulations and building codes that set minimum criteria for lighting design [12–16]. However, in the least developed countries, especially sub-Saharan Africa, this case is still not clear and lacks some research [17]. Both qualitative and quantitative aspects of workplace illumination are considered by many researchers as the key factors determining the employees' productivity. The working promptness, excellence, interruptions, truancy, and accident rate are all affected by the environmental lighting conditions [5, 18]. Moreover, for many years, light has always been known for its paramount functions of not only enhancing visual performance but also making the occupants feel more pleasant, more comfortable, colorful, stimulated, and less oppressed [19].

Visual discomfort and physiological and psychological strain such as anxiety, fatigue, lethargy, headaches, eyestrain, migraine, nausea, back pain, neck pain, shoulder pain, poor concentration or lack of mental alertness, and daytime sleepiness among VDT workers are primarily connected with inadequate lighting in the working place and in most cases decrease work performance and efficiency [20]. Thus, providing adequate or quality lighting condition in a working space goes beyond the act of just installing a suitable quantity of light. It involves many factors including illuminance uniformity, luminance distributions, light color, color rendering and color temperature characteristics, nature of light (natural or artificial), flicker, and glare control among others [21, 22].

The lighting illuminance level and uniformity refer to the maintained minimum average illumination required to accomplish a specific task in a given work plane [23]. Most of the lighting codes from different parts of the world such as the European standards (EN 12464) specify that, for the activities on the working plane such as writing, reading, and typing, the horizontal illuminance level should be maintained at a minimum of 500 lx. Additionally, it is advised that the illuminance level in the surroundings of the working plane up to a distance of half a meter should be maintained at 300 lx, whereas the remaining areas should be illuminated at 200 lx [23–25]. The nature of the light source, its position, and mounting height, luminaire type, and its light distribution determines the quantity, quality, and uniformity of illuminance in the workplace. Researches point out that right illuminance level and uniformity improve occupants' visual perception and decrease the signs of fatigue, including eye pain and headache. [23, 26]. Also, well-maintained illuminance levels increase occupants' mood and alertness (reduce sleepiness) which are essential factors for increasing occupants' performance [25].

The correlated color temperature (CCT) and color rendering index (CRI) of the light source dictate the way the light is perceived in a given space. Both CCT and CRI play a dynamic role in addressing the psychological and physiological functions of the occupant [27]. CCT of the light source influences visual perception and is significantly linked to visual satisfaction, mood, cognition, and comfort [23]. According to Meloy and McLeod [28, 29], cognition is a mental process of acquiring knowledge and understanding through thinking, knowing, remembering, judging, problem-solving, reasoning, comprehension, and attention. Thus, applying appropriate CCT in the working environment enhances occupants' motivation, improves health and cognition, increases working efficiency, and hence improves productivity. Existing researches have also identified that occupants subjected to daylighting working environments exhibit higher work satisfaction and excellent performance [30].

van Bommel [31] defines the glare as the sensation produced when one part of the visual field is much brighter than the average brightness to which the visual system is adapted. Glare existence in the working environment creates a veil of luminance that reduces the visibility of the target and can have direct or indirect interference with vision. The condition of glare where there is a direct interference with vision is known as disability glare and the condition where the vision is not directly interfered, yet discomfort, annoyance, irritability, or distraction is experienced is called discomfort glare. Disability glare in the working environment causes mental and physical tiredness while discomfort glare affects the individual's possibility to concentrate on the task [32] especially those individuals working on visually demanding tasks such as VDT [33].

Flicker occurs when light is modulated at lower frequencies. Flicker is common in fluorescent lamps operated on magnetic ballasts [31]. The human eye is sensitive to flicker, and its existence in the workplace cause physical discomfort, eyestrain, and visual fatigue [32]. The study by Wilkens et al. [34] identified that the occurrence of headache among occupants could be significantly lowered when electronic ballasts are used. In another study [35] regarding the impact of flicker on occupants' well-being, performance, and arousal Kuller and Laike identified that employing high-frequency ballasts instead of magnetic ones can limit flicker effect, reduce brain stress, and improve productivity. Several studies have also shown that lighting preferences differ significantly from one user to another based on their mood, activity, culture, health problem, and individual preferences [27, 36]. On the other hand, studies revealed that the freedom or autonomy of occupants to adjust the lighting of their workplaces according to their preferences has a positive effect on their work satisfaction, motivation, vigilance, and visual comfort [32, 36–38]. Additionally, a report by CIBSE [14] also indicated that a workspace which does not offer freedom of controlling the environment leads to increased discomfort and stress among occupants. Therefore, the user-centric lighting system is thought to improve occupants' satisfaction and comfort in modern office buildings.

A large amount of research has identified that lighting also exerts nonvisual effects on biological rhythms, commonly known as body circadian cycle [5, 39]. In the year 2002, David Berson noted that apart from traditional photoreceptors (rods and cones) typically responsible for vision, in mammals' retina, there is another photoreceptor called intrinsically photosensitive retinal ganglion (ipRGC) [20]. This is a part of the suprachiasmatic nuclear (SCN) of the hypothalamus [31, 40] which is a section of the brain that controls the body circadian cycle and is mostly involved in several nonimage forming functions such as promoting alertness, mood, cognitive performance, controlling body metabolism, DNA damage response, hormone production, and even cell cycle regulation and division [19,39–43]. Biological clock or circadian rhythms are found in all living organisms and is the body sleep-wake cycle commonly known as light-dark cycle [20, 44]. It is an intricate biological system which through light perception the brain informs the body on how to regulate various systems such as body temperature, release of hormones from pituitary gland, controlling body metabolism, sleep patterns, and secretion of hormones such as melatonin (sleep-regulating hormone) and cortisol (stress regulating hormone) [23, 45]. On experimental investigation of the relationship between disrupted circadian rhythmicity and disease, Lucassen et al. [44] concluded that a disrupted circadian rhythm reversibly induces detrimental effects on multiple human biological processes.

Light has a dual nature. It appears as both wave and particle, and the wavelength of light determines the color perceived by the eye. In the visible region which ranges from ≈380 nm to 780 nm [26], short wavelengths between 380 nm and 500 nm are considered as violet and blue light. The photons in this region have high energy, and it is known as high energy visible light (HEVL). The melanopsin photopigment contained in the IpRGC in the retina is more sensitive to short-wavelength visible blue light (*λ* ≈ 480 nm) present in the HEVL region [40, 46, 47]. The sunlight has a continuous spectrum and is rich in blue light. Thus, exposure to daylight provides an advantage of not only visual enhancement but also regulating the body's circadian circle and improving the occupants' well-being and productivity [40]. The advancement of lamp technology has proportionally altered the working culture, and working times have correspondingly increased. Exposure to artificial lighting both at night and during the day has also increased. Estimations show that over 75% of the world's population is exposed to light during the night [47], and night shift works are relatively high around the globe [44].

Furthermore, epidemiological evidence demonstrates that people working in night shift works have a higher rate of cancer cases and a high rate of breast cancer among the female population, especially in the industrialized countries [20]. Metabolic syndrome, osteoporosis, bone fractures decreased sleep quality, increased body weight, and a higher prevalence of cardiovascular disease [44] are also among health effects triggered by inadequate lighting exposure. Besides, the aggravated effect of the poor indoor lighting environment and circadian entrainment extends to body malfunction, which in most cases results in symptoms like daytime sleepiness, night-time insomnia, confusion, seasonal depression, gastrointestinal distress, irritability, increased error rate, memory disruption, and cognitive confusion [20]. The multiplying effect of these symptoms at a critical stage increases individuals' susceptibility to illness [48], mental and psychological discomfort, and job dissatisfaction and hence decreases productivity and work efficiency [32].

Although previous studies have clearly demonstrated that inadequate lighting environment in offices affects workers' well-being, work productivity, and efficiency, similar studies in developing countries especially in African countries are still rare. Thus, the primary objective of this study is to analyze the effects of the office building lighting quality on working efficiency of workers in Tanzania. The research is very significant in promoting awareness and providing a contribution to the existing body of knowledge regarding the effect of inadequate lighting environment on occupants' health, productivity, and working efficiency.

## 2. Materials and Methods

### 2.1. Field Survey

The study was conducted at MUST in Tanzania from June to September 2018. In order to elucidate the lighting scenario and its effect on occupants, four representative offices were selected based on their unique characteristics and representation merits. Their floor plans and physical features were studied as demonstrated and summarized in Table 1 below. Illuminance levels were measured, average illuminance in each room was determined, and the uniformity ratio was calculated. Structured questionnaires were also administered to randomly sampled staff members through an online questionnaire tool.

### 2.2. Characteristics of the Study Area

MUST is one of the oldest educational institutions in Tanzania. It was founded in 1986 as Mbeya Technical College (MTC) offering full technician certificate (FTC) education to less than 500 young Tanzanians under the Russian training support. “*The facility was constructed and equipped under the technical cooperation agreement between the Government of the United Republic of Tanzania (URT) and the Government of the former Union of Soviet Socialist Republics (USSR)*” [3]. To achieve the dream of the founder, the institution was upgraded to become Mbeya Institute of Science and Technology (MIST) in 2005. In 2012, upon fulfilling the minimum requirements for university accreditation by Tanzania Commission for Universities (TCU), MIST was accredited as a university, and it was renamed Mbeya University of Science and Technology (MUST) [2]. Throughout all these transformations, very few infrastructural changes and increment from the existing ones were done, despite the exponential increment of the number of students and staff members. The demand to accommodate the increased number of occupants in the existing buildings has seen the alteration of many indoor open spaces, transition spaces, stores, basement spaces, and assembly points into classrooms and staff's offices. These alterations have necessitated the existence of windowless rooms and spaces with little natural light, much dependencies on artificial lighting, and even inadequate artificial lighting environment.

### 2.3. Illuminance Distribution Measurements

**Figure 1:**
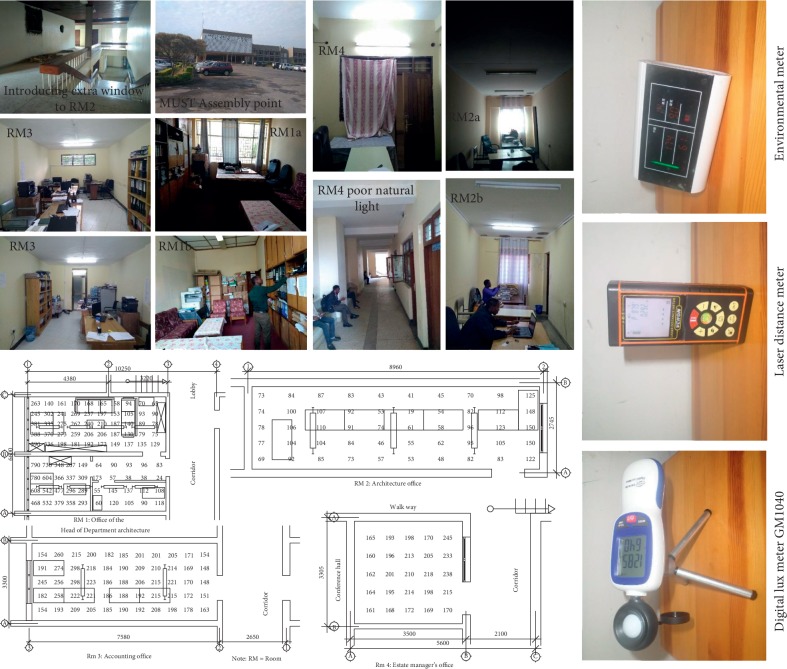
The floor plans of the representative offices and images showing the impression of lighting set up and the equipment involved in measurements.

The horizontal illuminance values and distribution in the selected four representative offices and indoor air quality in each office were recorded using a digital lux meter GM1040 mounted on the unique tripod stand at 0.85 m from the floor and electronic environmental meter. The room dimensions were measured using a laser distance meter, and the illuminance grid points were determined based on the room dimensions, as shown in Figure 1 below. The workplace illuminance uniformity in each of the surveyed office was determined according to the following equation:

(1) $$\begin{array}{c} U_{R}=\frac{{\text{Eh}}_{\text{min}}}{{\text{Eh}}_{\text{avg}}}, \end{array}$$

where Eh_min_ is the minimum desk illuminance and Eh_avg_ is the average desk illuminance [49]. The value obtained was compared to the CIBSE guidelines, which recommend a minimum uniformity ratio of 0.8 [50].

### 2.4. Survey Method and Questionnaire Design

A set of the structured questionnaire was administered to respondents to establish their rate of satisfaction, feeling about the lighting environment, the existence of both visual and nonvisual problems in their workplace, and how these factors influenced their health and working efficiency. The questionnaire was therefore broken down into three parts, including nine questions on demographic information, twenty-eight questions about the perceived lighting quality, feelings, satisfaction, and visual annoyance, and ten questions about health issues related to lighting problems in the working environment. A Likert five-point scale system and a dichotomous two-scale system of “Yes and No” questions were applied in structured questions. Additionally, two open-ended questions were included to capture in-depth the lighting problems the users are facing in their offices. Their comments, feelings, lighting preferences, and improvement measures were also assessed. A pilot study was conducted online from March to June 2018 to test the effectiveness of the intended online questionnaire method. Twenty Ph.D. and Master's degree students in different job categories from MUST were involved in the study. The feedback on the professionalism of the used language, length, and other ambiguities of the questionnaire were issued for the final version which was used in the survey. A simplified language was used in the questionnaire, and a short introduction was given to every section of the questionnaire for more clarity and simplicity.

### 2.5. Variable Definitions

All variables used in this questionnaire were primarily extracted from the existing research in most of the literature covered above. The respondents were asked several questions in order to self-evaluate themselves and report on their perceived lighting quality. Questions on illuminance level and distribution, visual comfort-ability, lighting reflections, brightness and color contrasts, glare existences and user's perception, flicker existence, sound annoyance, and concentration or alertness were asked. Respondents were also asked to rate the attractiveness of the surrounding luminous environments and their level of satisfaction. Additionally, respondents were asked to self-report the prevalence of musculoskeletal symptoms [11], visual symptoms, and systematic symptoms [51]. Issues related to dry eyes, teary eyes, eyestrain, blurred vision, itchy eyes, shoulder pain, neck pain, back pain, headache, and stress were asked. Sleeping pattern at night, especially on work-days, was also assessed in the questionnaire. All variables covered in the questionnaire were predominantly structured and grouped under four main questions which were as follows:

- *Is there any discomfort caused by the lighting environment in your office/workplace?* The question aimed at assessing the quality of the luminous environment and its comfort ability to the occupants.
- *How do you think this lighting influences your working efficiency/performance?* Seeking to measure the extent of the influence of the luminous environment on occupants' working efficiency.
- *How do you think this lighting affect your well-being?* Aiming at assessing the effect of the existing luminous environment on occupants' health.
- *What should be done to improve the situation?* Aiming at capturing the occupants' views on how the situation can be made better and their preferences.

### 2.6. Respondents

All staff members (about 600 employees) at MUST were the target group in this research. A simple random sampling method was adopted in questionnaire distribution. The target sample of respondents was 240 people, determined by using Taro Yamane's formula in the following equation:

(2) $$\begin{array}{c} n=\frac{N}{1+Ne^{2}}, \end{array}$$

where “*n*” is the minimum number of respondents required (sample size), “*N *” is the study population size, and “*e*” is the level of precision or acceptable sampling error assumed at 95% confidence level (alpha level of 0.05) [52, 53]. The questionnaire URL was administered to 252 randomly selected staff's from a list of 600 employees at MUST. Staffs emails and social platforms such as WhatsApp and Wechat were used in questionnaire distribution. A total number of 171 responses were received back, constituting a response rate of 67.32%.

### 2.7. Data Management and Analysis

All the data were evaluated and analyzed in the statistical package for social science (SPSS version 20), and some graphs were treated using an excel spreadsheet. Descriptive statistics tools such as percentages, frequencies, and graphical representation were applied to analyze the quantitative information. Cross tabulation and statistical tests such as one-sample *T* test and bivariate correlation were also used in order to show the confidence intervals and the association between variables. Pattern matching technique was also applied to qualitative data, whereby the collected information was technically analyzed under the groups with similar meaning. Triangulation of the research findings was finally done to conclude.

## 3. Results

### 3.1. Respondents' Demography

In this study, among the 171 respondents, 39.8% were female and 60.2% were male. The academic staff constituted 60.8% of all respondents, while 22.2% were management staffs, 12.3% logistics, and 4.7% were from other work categories. Age issues were also considered whereby all age groups were covered in the survey. In this regard, 12.9% of all respondents were aged between 20 and 30 years, 77.2% were aged 31–45, and 9.9% were between 46 and 60 years old. Only 2.9% of the respondents were Ph.D. holders, 21.1% were vocational and diploma certificate holders while 33.33% and 42.7% were bachelor and master's degree holder, respectively. Furthermore, 97.7% of all respondents reported that they have been working at MUST for less than 20 years, and 2.3% have worked at MUST for more than 20 years. 80% of the respondents reported having a 5-day work per week, and the average working hours for all participants were 7.6 hours per work-day. Among all respondents, only 13.5% had a vision impairment, and most of the time, they used glasses due to myopia. Additionally, 76.6% (*n* = 131) of all respondents reported that they spend between 4 and 9 hours working on the computer.

### 3.2. Lighting Quality and Suitability

Five questions were inquired of respondents to self-report their measure of perceived lighting quality and its suitability by using a five-point Likert scale ranging from 1-strongly disagree to 5-strongly agree. The descriptive statistics evaluation and one sample *T* test revealed that the majority of respondents 60.2% (*M* = 2.40; SD = 0.92; *t* = 34.25; CI = 2.26–2.54) thought that the illuminance level and its distribution in their workplace were inadequate. Visual comfort level was also regarded as uncomfortable by 50.3% (*M* = 2.53; SD = 0.84; *t* = 39.65; CI = 2.41–2.66) of all respondents, and the majority of respondents 56.1% (*M* = 2.97; SD = 0.70; *t* = 55.23; CI = 2.86–3.07) reported that the lighting condition in their workplace created an environment where they could reasonably perform their detailed tasks precisely. Furthermore, the internal lighting reflection was voted as poor by 43.9% (*M* = 2.74; SD = 0.83; *t* = 43.11; CI = 2.61–2.86) of all respondents, whereas the brightness and color contrasts were perceived as acceptable by 56.1% (*M* = 2.49; SD = 0.89; *t* = 36.59; CI = 2.36–2.63).

Also, the relationship between variables was tested using Pearson's coefficient at a confident level of 95% (*P* value 0.05). The results depicted a positive moderate relationship between variables and *P* values <0.05, signifying that the variables are reasonably correlated and statistically significant, as shown in Table 2. Moreover, the lighting illuminance was also physically measured in four representative offices, and the uniformity was calculated, as shown in Table 3.

From Table 3, it can be identified that the illuminance level in all offices is below lamp technology, inadequate lamp spacing, incorrect lamp mounting height, dirtiness around lamp luminaries, and poor choice of partitioning materials in some offices and poor reflectivity of walls, floors, and ceiling surfaces.

### 3.3. Visual Comfort and Satisfaction

Occupants were asked to self-report the level of comfort created by the lighting environment in their workplace by using a five-point Likert scale of agreement ranging from 1-low agreement to 5-high agreement. The results indicate that a small number of the respondents (46.2%) (*M* = 3.06; SD = 1.23; *t* = 32.63; CI = 2.87–3.24) experienced glare problem which they could not resolve easily due to the absence of window curtains and other forms of shading devices. Observation depicted that, in some offices which had no direct sunlight, the reported glare was due to the inadequate installation of artificial light fixtures. Furthermore, the poor choice of furniture and poor furniture ergonomics was other factors which contributed to glare problems in the workplace, especially to the occupants who worked on VDT. Visual destruction and annoyance exerted by lighting flicker (*M* = 2.97; SD = 1.28; *t* = 30.22; CI = 2.77–3.16) and annoying sound (*M* = 2.84; SD = 1.36; *t* = 27.40; CI = 2.64–3.05) from defected circuits also influenced the occupants' visual comfort and satisfaction. Moreover, 50.9% (*M* = 2.67; SD = 0.99; *t* = 35.19; CI = 2.52–2.82) of the respondents reported that their luminous environment was not attractive, 56.1% (*M* = 3.07; SD = 1.27; *t* = 31.56; CI = 2.88–3.26) thought that it does not promote alertness, and 58.5% (*M* = 2.64; SD = 1.13; *t* = 30.49; CI = 2.47–2.81; *P* < 0.01) agreed that it significantly affected their work efficiency.

The subjective assessment of the daytime alertness and concentration among the subjects also revealed that 49.7% (*M* = 2.68; SD = 0.88; *t* = 39.85; CI = 2.55–2.81; *P* < 0.01), 36.8% (*M* = 2.94; SD = 1.03; *t* = 37.44; CI = 2.79–3.10; *P* < 0.01), and 38% (*M* = 3.11; SD = 1.09; *t* = 37.24; CI = 2.94–3.27; *P* < 0.01) of the respondents encountered unexpected tiredness, sleepiness, and losing concentration, respectively. Generally, majority of the respondents (56.2%) (*M* = 2.56; SD = 1.04; *t* = 32.10; CI = 2.40–2.71; *P* < 0.01), were less satisfied with their office's lighting quality, and nearly half of the respondents (49.7%) (*M* = 2.76; SD = 1.03; *t* = ; CI = 2.60–2.92; *P* < 0.01) evaluated it as uncomfortable. The ANOVA test also depicted a statistically significant interaction between lighting quality and occupants' comfort and satisfaction in the surveyed office building F (16, 58.67) = 2.073, *P*=0.012.

### 3.4. Influence of the Lighting Environment on Occupants' Well-Being

**Figure 2:**
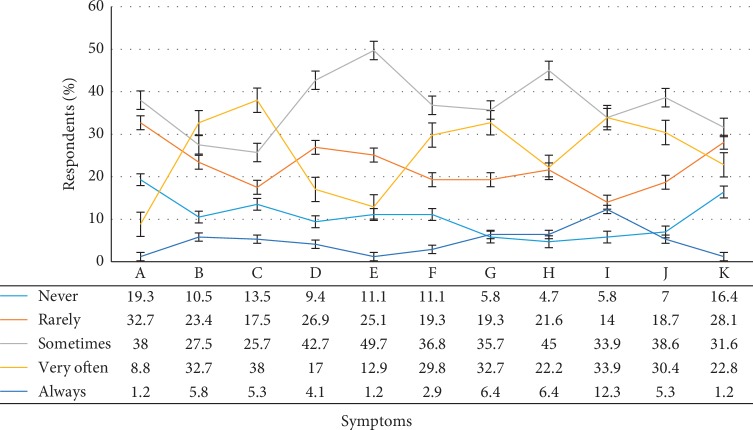
The perceived influence of lighting environment on occupants' health. (A) Insomnia. (B) Back pain. (C) Neck pain. (D) Shoulder pain. (E) Tiredness. (F) Sleepiness. (G) Teary eyes. (H) Headache. (I) Eyestrain. (J) Eye fatigue. (K) Stress.

The subjective assessment using structured questionnaire under five-point Likert scale ranging from “1 = *never*, 2 = *rarely*, 3 = *sometimes*, 4 = *often*, and 5 = *always*” was employed to determine the influence of lighting environment on occupants' health. The descriptive statistics analysis and one-sample *T* test revealed that 45% (*M* = 3.04; SD = 0.94; *t* = 42.23; CI = 2.90–3.18), 33.9% (*M* = 3.33; SD = 1.05; *t* = 41.41; CI = 3.17–3.49), and 35.8% (*M* = 3.15; SD = 1.00; *t* = 41.22; CI = 3.00–3.30) of the respondents occasionally experienced headache, eye-strain, and teary eyes during working, respectively. Likewise, 38.6% (*M* = 3.08; SD = 0.99; *t* = 40.68; CI = 2.93–3.23) of respondents reported eye fatigue. Also, 31.6% (*M* = 2.64; SD = 1.04; *t* = 33.11; CI = 2.49–2.80) of the respondents agreed that no matter how much work they had, they sometimes felt stressed, and 38% (*M* = 2.40; SD = 0.94; *t* = 33.50; CI = 2.26–2.54) agreed that they occasionally suffered insomnia during night-time. Moreover, some respondents reported suffering from musculoskeletal symptoms such as shoulder pain 42.7% (*M* = 2.80; SD = 0.97; *t* = 37.70; CI = 2.65–2.94), neck pain 38% (*M* = 3.04; SD = ; 1.15 *t* = 34.74; CI = 2.87–3.21), and back pain 32.7% (*M* = 3.00; SD = 1.11; *t* = 35.47; CI = 2.83–3.17) when working in their offices as illustrated in Figure 2.

## 4. Discussion and Recommendations

For the past few decades, the advancement in information communication technology has changed the landscape of offices around the world. The wide use of VDT in the workplace has called for new office design requirements. Awareness of this scenario has been improved, and remedial actions are widely practiced in most of the developed countries and fast-developing countries. However, in most of the least developed countries, especially African countries such as Tanzania, this case is still not clear and lacks some research. Reports have indicated that poorly designed offices environments such as inadequate lighting affects human health and work performance. Based on that, this research aimed at analyzing the effects of lighting quality on working efficiency of workers in an office building in Tanzania. One office building at Mbeya University of Science and Technology was selected as a case study. Physical observation and questionnaire survey methods were employed to collect the data. The results have demonstrated that the illuminance level and uniformity in this particular workplace were inadequate and to some extent and affected the occupants' health, work efficiency, and productivity.

Moreover, the research identified that most of the office had glare problems, flicker, and sometimes, and the lighting luminaries produced some annoying sounds. Studies assert that the presence of these problems in the working environment significantly reduces visual acuity, exerts difficulties in performing the tasks, and causes visual symptoms such as headache, eyestrain, teary eyes, and visual fatigue [54]. Some other literature affirms that the inadequately provided lighting environment also leads to mental and physical tiredness, affects the individual's possibility to concentrate on the task [32], reduces the workers' vitality, and induces sleepiness during work-times. Furthermore, the increased accidents rate, work dissatisfaction, and other forms of discomfort are also linked with insufficient lighting in the workplace [20].

Improving occupants' satisfaction requires an integrated lighting design method. A design which gives occupants the freedom to control some aspects of their offices' lighting environment. User-centric lighting design is an ideal solution for improved occupants' health and sustained good performance. This kind of lighting design was also reported in [6, 41] as being an efficient method of increasing occupants' level of comfort, work environment satisfaction, and improved work efficiency. In order to achieve this design in the Tanzania context, the research recommends that an ambient-task lighting method should be adopted because it offers the advantage of providing illumination where it is needed most, and it is more economical. Furthermore, ambient-task lighting method allows the individual worker to adjust the appropriate illuminance for the task being performed according to their preferences. Moreover, this research recommends that efforts should be made to provide windows to some offices wherever possible because access to daylight and views to the external environment is beneficial to occupants.

Besides, the existing literature affirms that occupants strongly prefer daylight to electric lighting [30]. Daylighting is considered the best source of light with exceptional color rendering and continuous spectrum, which offers the best light for human visual comfort and health [7]. Access to windows increases work environment satisfaction, provides information about surroundings and weather, reduces occupants' discomfort, and improves health and performance [6]. Additionally, with the wide adoption of VDT working tools, the provision of sustainable office lighting environment is inevitable [55]. Thus, the lighting in the working environment should be designed to provide not only the right visual conditions but also the condition which promotes occupant's well-being. Because of that, this research recommends that creating a lighting condition which provides for the needed level of visual performance, improves spatial appearance, enhances safety, and contributes to improving occupants' well-being should be mandated in all workplaces. Additionally, the Occupational Safety and Health Administration (OSHA) should set and implement the occupational health legislation and regulations which will include the minimum standards for offices lighting and ergonomics compliance assessment criterion to be adopted by all VDT workplaces and office construction developers in Tanzania.

## 5. Conclusions

Providing a healthy working environment is now a hot topic. Scores of the literature have reported that poor indoor environmental quality such as lighting has a detrimental effect on human health, and in case of the office working population, it affects their work performance as well. This study has examined the effects of lighting quality on working efficiency of workers in an office building in Tanzania. Some offices from the administration building at Mbeya University of Science and Technology were examined. The results have indicated that workers are less satisfied with their offices' lighting environment. Physical measurements and researchers' observations also depicted that the quality of the illumination was insufficient for a healthy working environment. Visual destructions such as glare discomfort, lighting flicker, and annoying sounds from defected circuits were alarming, and in most of the cases affected the occupants' concentration. Also, the occupationally induced health symptoms such as eyestrain, headache, teary eyes, back pain, shoulder pain, and neck pain were significantly reported.

Furthermore, the research identified that poor lighting design techniques, use of outdated lighting technology, not adhering to different task lighting requirements, absence of local lighting codes and governing regulations, poor use of the concept of room index ratio, and poor choice of lighting luminaries were the factors which contributed to inadequate lighting in the surveyed office building (Table 4). Other factors were the poor periodic maintenance and services of the lighting systems, which resulted in the accumulation of dirtiness around the luminaries and dilapidation of the lighting infrastructures. On the other hand, this research considers that the reported musculoskeletal pain among respondents could also be linked with poor ergonomic practice in the workplace especially those VDT operators. Therefore, this research recommends for further investigations about the appropriateness of the offices' physical environment in Tanzania. Lastly, besides the given remedy for the observed lighting design defects in the study area, this research also recommends that the government of Tanzania should foster the process of setting and enacting local construction codes to govern and regulate the construction industry so as to safeguard its citizens from emerging poor built environment and unhealthy work environment.

## 6. Study Limitations and Direction for Future Research

This research was limited to analyzing the effects of lighting quality on working efficiency of workers in an office building in Tanzania. The factors contributing to the effects have been identified and analyzed through physical observation and questionnaire survey. Four office rooms with good representation were identified, and measurements and physical analysis were conducted. However, the separation between sunlight and artificial light was not clearly made during illuminance measurements. Thus, prediction of the illuminance contribution between the two cannot be made. Some offices received a considerable amount of sunlight; however, occupants still kept the artificial light on to compensate the insufficiency. Therefore, in future research, it is recommended that these two scenarios be done separately in order to make a comparison and establish which kind of light is inadequately provided. Furthermore, the illuminance level requirement differs from one person to another and from task to task. The results of this study could be influenced by the characteristics of the studied population sample. The workers in the surveyed building have a variety of works performed in their respective offices, and their lighting requirements are different. Thus, for future studies, we recommend that groups of similar lighting requirements should be studied separately.

## Figures and Tables

**Table 1:** A summary of the offices' physical characteristics and lighting condition in the surveyed offices.

Office	Function	Parameters	Physical characteristics
1	Office of Head of Department (Architecture) located in the first floor	Ceiling	Plasterboards at 3.485 m above floor painted white and decorated to matt finish hardwood
		Walls	Plastered concrete blocks painted off white
		Floor	Red carpet
		Working plane	Matt finish hardwood table, close to the window, at 0.714 m above the floor
		Lighting characteristics	Both naturally and artificially lit; clear glass on the window with translucent curtain, four ceiling-mounted fluorescent tubes TU and T8, 36 W, fixed on magnetic ballasts
		Humidity and temperature	The humidity was 65%, and the temperature was 23°C

2	Staff's office (Architecture Department) located in the second floor	Ceiling	Gypsum board, painted in white color
		Walls	Plastered concrete block painted in white color
		Floor	Cement sand screed
		Working plane	Mixed type of furniture, ergonomics not well adhered to
		Lighting characteristics	Both naturally and artificially lit, a small side window with clear glass and a translucent curtain, three surfaces mounted fluorescent tube Philips T8, 36 W fixed on a magnetic ballasts
		Humidity and temperature	The humidity was 70%, and the temperature was 25°C

3	Accounting office located in the basement	Ceiling	Concrete soffit painted white at 3.213 m headroom
		Walls	Plastered and waterproofed concrete block, painted in white color
		Floor	White ceramic tiles
		Working plane	Matt and gloss finish hardwood tables at 0.75 m above the floor
		Lighting characteristics	Artificially lit, with a ventilation window at ceiling level supplied with mosquito gauze, two fluorescent tubes TU and T8, 36 W, fixed on magnetic ballasts and mounted on ceiling soffit
		Humidity and temperature	The humidity was 75%, and the temperature was 21°C

4	Estate manager's office located in the first floor	Ceiling	Gypsum board at 2.8 m from the floor
		Walls	Concrete blocks, plastered and painted off-white
		Floor	Cement sand screed
		Working plane	Matt and gloss finish hardwood tables at 0.75 m above the floor
		Lighting characteristics	Both naturally and artificially lit; clear glass on the window with translucent curtain, one pendant fluorescent tubes TU and T8, 36 W, fixed on magnetic ballasts mounted on the wall at ceiling level
		Humidity and temperature	The humidity was 66%, and the temperature was 23°C

**Table 2:** The correlation coefficients and significance levels between the variables for the lighting quality and its suitability.

Variables		L&D	VC	Pr	IR	Br&C
Lighting level and distribution (L&D)	Pearson's correlation	1				
	Sig. 2-tailed					
	*N*	171				

Visual comfort (VC)	Pearson's correlation	0.532^*∗∗*^	1			
	Sig. 2-tailed	0.0001				
	*N*	171	171			

Precision (Pr)	Pearson's correlation	0.196^*∗*^	0.333^*∗∗*^	1		
	Sig. 2-tailed	0.010	0.0001			
	*N*	171	171	171		

Internal reflection (IR)	Pearson's correlation	0.179^*∗*^	0.280^*∗∗*^	0.448^*∗∗*^	1	
	Sig. 2-tailed	0.019^*∗*^	0.0001	0.0001^*∗∗*^		
	*N*	171	171	171	171	

Brightness and color contrast (Br& C)	Pearson's correlation	0.195^*∗*^	0.279^*∗∗*^	0.310^*∗∗*^	0.335^*∗∗*^	1
	Sig. 2-tailed	0.011^*∗*^	0.0001	0.0001	0.0001	
	*N*	171	171	171	171	171

^*∗*^The correlations which are significant with respect to *α* = 0.05. ^*∗∗*^The correlations which are significant with respect to *α* = 0.01.

**Table 3:** The measure average offices illuminance level and uniformity.

Office	Average horizontal task illuminance level (lux)		Horizontal illuminance uniformity ratio (Eh_min_/Eh_avg _)	Specified minimum task illuminance and uniformity
RM1	Head of the department	196	0.332	500 lux for the tasks involving writing, reading, and typing, and 0.8 of the illuminance uniformity ratio
	Assistant Head of the department	443	0.336	
	Secretary office	105	0.524	
RM2	84		0.488	
RM3	198		0.747	
RM4	193		0.834	

RM = room; Eh_min_ = minimum desk illuminance; Eh_avg_ = average desk illuminance.

**Table 4:** Summary of the identified spatial lighting problems and the recommended remedial.

Factors identified	Identified problem	Proposed solution
Illuminance level and spatial partitioning materials	Lighting on the work plane was below the recommended level	Lighting fixture should be mounted at the appropriate height to ensure proper distribution of illuminance
	Lamp spacing and mounting height against the room index ratio was inconsistent	Lamps and luminaires should be cleaned regularly
	Uneven distribution of light on the working area and the surrounding was observed	Defected lamps should be replaced
	Veiling reflection was identified	Room surfaces reflectance should be improved by painting the walls with light colors, e.g., Paint white
	Light being wasted to the ceiling and other areas which are not the visual target	Ambient task lighting methodology should be adopted to localize and personalize lighting
	Using lamps luminaries with the reduced light output	Provide lighting fixtures and lamps with more efficient light output, e.g., T5 or LED lighting technology
	Poor choice of partitioning materials and partitioning style	Lamps should be mounted on the luminaries with excellent light outputs
		Use transparent or translucent partitioning materials, e.g., glasses; also, the partitioning should be kept a minimum

Brightness and contrasts	Luminaries were dully covered with cobwebs and other black spots, and bared lamps or surface mounted lamps were common	Light controllers should be installed to direct light to the required task area
	Reflecting surfaces such as walls, ceiling, and floors were painted to colors with poor reflective index	Luminaries with more efficient light output should be used
		Reflecting surfaces should be painted into more bright colors, e.g., white colors for more reflection of light
		Use lamps with good color rendering effect

Flicker and sound	Lighting in most of the office experienced intensified flicker and produced the annoying sound	Electrical circuits should be revisited, and all possible faults should be rectified
	Magnetic ballasts were widely used instead of using electronic ballasts	High-frequency control gears should be used, and all lamps near the end of their life should be replaced

Public awareness	The general public is not well informed about the effects of inadequate lighting on working efficiency and human health	Government and other stakeholders should prepare the education programs to raise awareness

Policy problems	Absence of local building codes and guiding regulations	Formulate and enact the local lighting design and building construction codes and regulations to guide the construction industry in Tanzania

## Data Availability

Data will be made available from the primary author upon request.
